# Severe infections due to *Francisella tularensis ssp. holarctica* in solid organ transplant recipient: report of two cases and review of literature

**DOI:** 10.1186/s12879-019-3863-0

**Published:** 2019-03-08

**Authors:** Olivier Bahuaud, Cécile Le Brun, Thomas Chalopin, Marion Lacasse, Julien Le Marec, Clémence Pantaleon, Charlotte Nicolas, Louise Barbier, Louis Bernard, Adrien Lemaignen

**Affiliations:** 1 Department of Internal Medicine and Infectious Diseases, University Hospital of Tours, Hospital Bretonneau, 2, boulevard Tonnellé, 37044 Tours Cedex 9, France; 2 François Rabelais University, Tours, France; 3 Department of Bacteriology, University Hospital of Tours, Tours, France; 4 Department of Cardiology, University Hospital of Tours, Tours, France; 5 Department of Hepatology, University Hospital of Tours, Tours, France; 6 Department of Digestive Surgery, Hepatobiliary Surgery and Liver Transplantation, University Hospital of Tours, Tours, France

**Keywords:** *Francisella tularensis*, Solid organ transplant, Pneumonia, Molecular diagnosis, Prevention

## Abstract

**Background:**

Tularemia is a rare zoonotic infection caused by bacterium *Francisella tularensis.* It has been well described in immunocompetent patients but poorly described in immunocompromised patients notably in solid organ transplant recipients.

**Case presentations:**

We report here two cases of tularemia in solid organ transplant recipients including first case after heart transplant. We also carried out an exhaustive review of literature describing characteristics of this infection in solid organ transplant recipients.

## Discussion and conclusions

Tularemia should be considered in case of pulmonary symptoms in solid organ transplant recipient with persistent fever. Molecular techniques might improve diagnosis and should be resorted to in cases of challenging diagnosis. Simple care and precaution tips should be given to the patients such as wearing masks in case of exposure.

## Background

Tularemia is an infrequent bacterial disease caused by Gram negative coccobacillus *Francisella tularensis*. The annual mean incidence in France is 0.07 cases per 100.000 inhabitants, rising up to between 0.16 and 0.20 per 100.000 inhabitants in the “Centre” region where our hospital is located [1]. In the European Union, the notification rate for 2016 was 0.2 cases per 100,000 inhabitants [2].

Clinical presentation depends on route of entry. Handling of infected animals and inoculation by infected arthropods are mainly associated with the ulceroglandular form, characterized by an ulcer with localized lymphadenopathy. Typhoidal disease is an acute form characterized by fever and symptoms of systemic infection that can lead to organ failure and septic shock [3]. The respiratory form is usually associated with the inhalation of aerosolized particles spread by infected rodents [3]. Approximately 20% of patients with Tularemia have been found to exhibit pulmonary involvement [4].

Few cases have been reported in immunocompromised patients and this infection is probably underrecognized due to atypical presentation and the fastidiousness of *F. tularensis* culture recovery [5–7].

We report here two new cases of Tularemia in solid organ transplant (SOT) recipients and present a review of literature.

## Case presentations

### Patient 1

A 64-year-old heart-transplanted patient was admitted in our department in May 2016 presenting with fever, chills and night sweats for 2 weeks. Laboratory results showed leukocytosis with neutrophilia and elevated C-reactive protein. The patient was receiving immunosuppressive therapy since transplantation in 2013. On admission, his treatment included prednisolone 1 mg/day, cyclosporin 80 mg b.i.d and mycophenolate mofetil 1500 mg b.i.d.

The patient developed an unproductive cough and progressive respiratory distress. Microbiological testings including blood cultures, DNA testing on serum for Epstein Barr virus, Cytomegalovirus, *Toxoplasma*, and on nasopharyngeal aspiration for *Mycoplasma pneumoniae* and *Chlamydophila pneumoniae* as well as serology for *Coxiella*, *Bartonella*, *Rickettsia* and *F. tularensis* were negative. Immunological investigations including ANA and ANCA were also found to be negative. Transthoracic echocardiography showed no sign of endocarditis, and a thoracic CT-scan revealed a pleural effusion and mediastinal lymphadenopathies without parenchymal impairment. Pleural liquid was exudative with negative cultures and cytology showed no sign of malignancy. A lymphadenopathy biopsy revealed necrotic tissue of undetermined significance.

The patient received several lines of antibiotic treatment: ceftriaxone and metronidazole, pyrimethamine and sulfadiazine replaced by cotrimoxazole due to renal function degradation. However, none of these therapies were clinically successful.

**Fig. 1:**
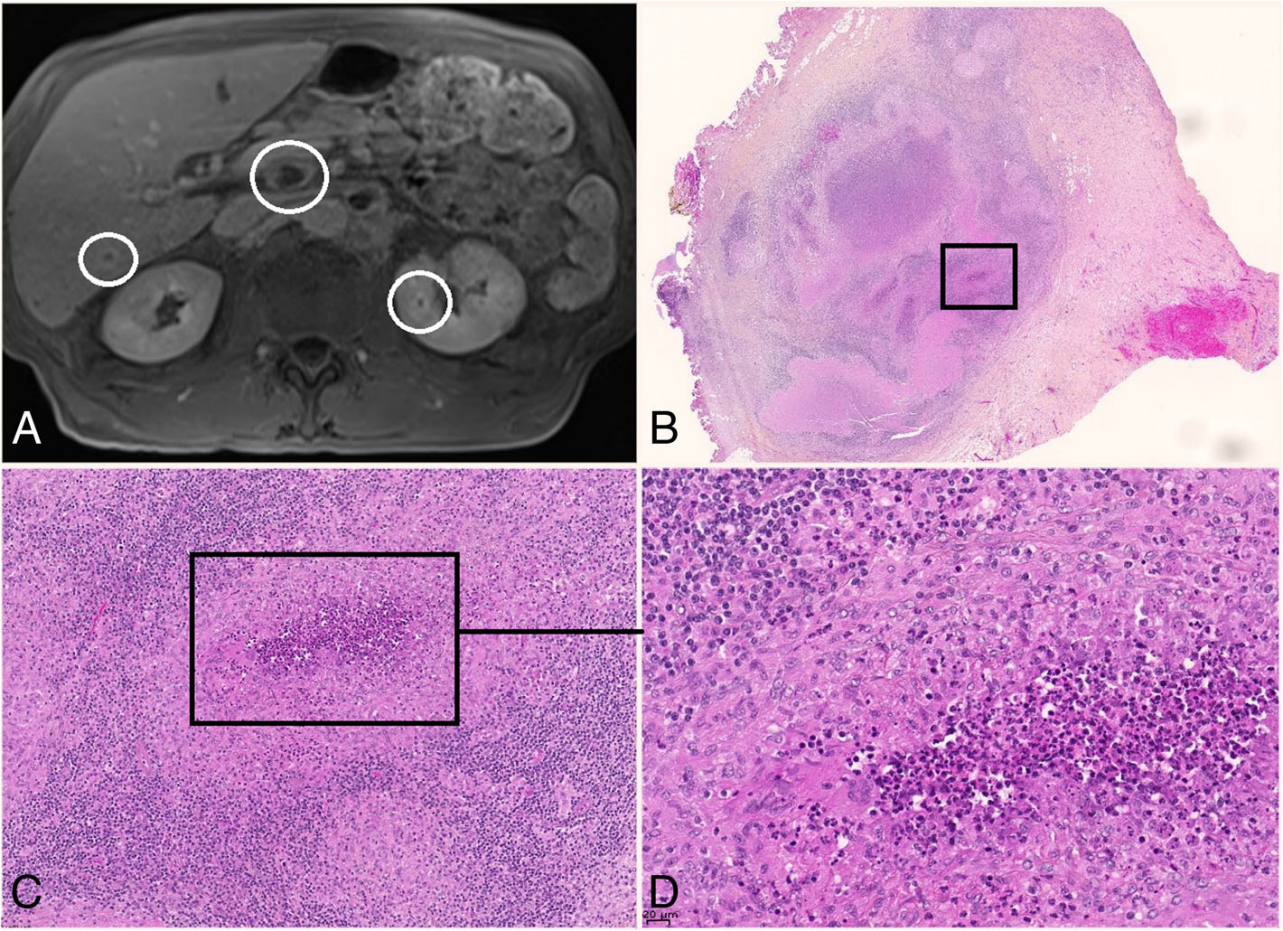
Celio-mesenteric adenopathy in systemic form of tularemia shown on MRI and histological slides. **a** Celio-mesenteric adenopathies on MRI. **b**, **c** and **d** nodular abcedal lymphadenitis on a celiac lymphadenopathy. HES coloration

After 4 months of evolution, he was re-admitted in our unit for fever and worsening of his general condition with body weight loss of 10 kg. Repeated microbiological investigations, including fungal serodiagnostic tools, gave negative results. PET-scan showed a hypermetabolism of mediastinal and celiomesenteric lymphadenopathies and a pulmonary parenchymatous lesion with a subpleural, paracardiac nodule. Both biopsies showed necrotic tissue with sputum but no germs and no sign of malignancy. Hepatic ultrasound followed by hepatic MRI revealed abdominal lymphadenopathies, and hepatic nodules. An exploratory laparotomy with lymphadenectomy was performed. Lymph node histology revealed a nodular lymphadenitis with pyogenic granuloma without caseum (Fig. 1). DNA was extracted from each sample using blood and tissue QIAamp kits (Qiagen EZ1), following the instructions of the manufacturer. Samples were frozen and stored at − 20 °C until analysis. We performed an in-house PCR using ‘Tul4R’ and ‘Tul4F’primers that specifically target the *tul4* gene of *F.tularensis* as described by Versage et al. [8]. The amplified sequence was 91 bp in length. The PCR mix contained 12.5 μl Premix Ex Taq (Takara), 4.5 μl water, 0.20 μl each of sense and anti-sense primers (25 μmol l-1 each), and 2.5 μl Sybr-Green for a total volume of 20 μl. DNA (5 μl) was added to the mix to obtain a total volume of 25 μl. Real-time PCR was performed with a Smart Cycler (Cepheid) using a program consisting of 600 s at 95 °C, 37 cycles of 60 s at 95 °C, 60 s at 60 °C, and 60 s at 72 °C, and a ramp up from 60 to 95 °C by 0 .2°C s-1 to obtain a melting peak curve. Beta-globulin detection was performed on all samples to test for the presence of inhibitors. This in-house PCR was positive for two lymph node biopsies (hepatic and paracardiac). Culture of the hepatic lymph node biopsy allowed the recovery of *Francisella tularensis ssp holarctica*.

The patient received combined antibiotic treatment with ciprofloxacin 750 mg b.i.d. and gentamycin 300 mg for 7 days followed by ciprofloxacin alone for 14 days. This treatment led to a full recovery without complication after 2 years of follow-up. His immunosuppressive therapy remained unchanged and no rejection event has been detected. Further questioning revealed an exposure to aerosolized pathogens during renovation work without airway protection in an old attic containing rat corpses.

### Patient 2

In February 2015, a 51-year-old patient was hospitalized in ICU for severe acidoketosis and septic shock with acute respiratory distress syndrome. He was treated for diabetes and hypertension and had received a liver transplant for mixed alcoholic and viral cirrhosis in 2008. His treatment included tacrolimus 0 .5mg and mycophenolate mofetil 500 mg twice a day.

**Fig. 2:**
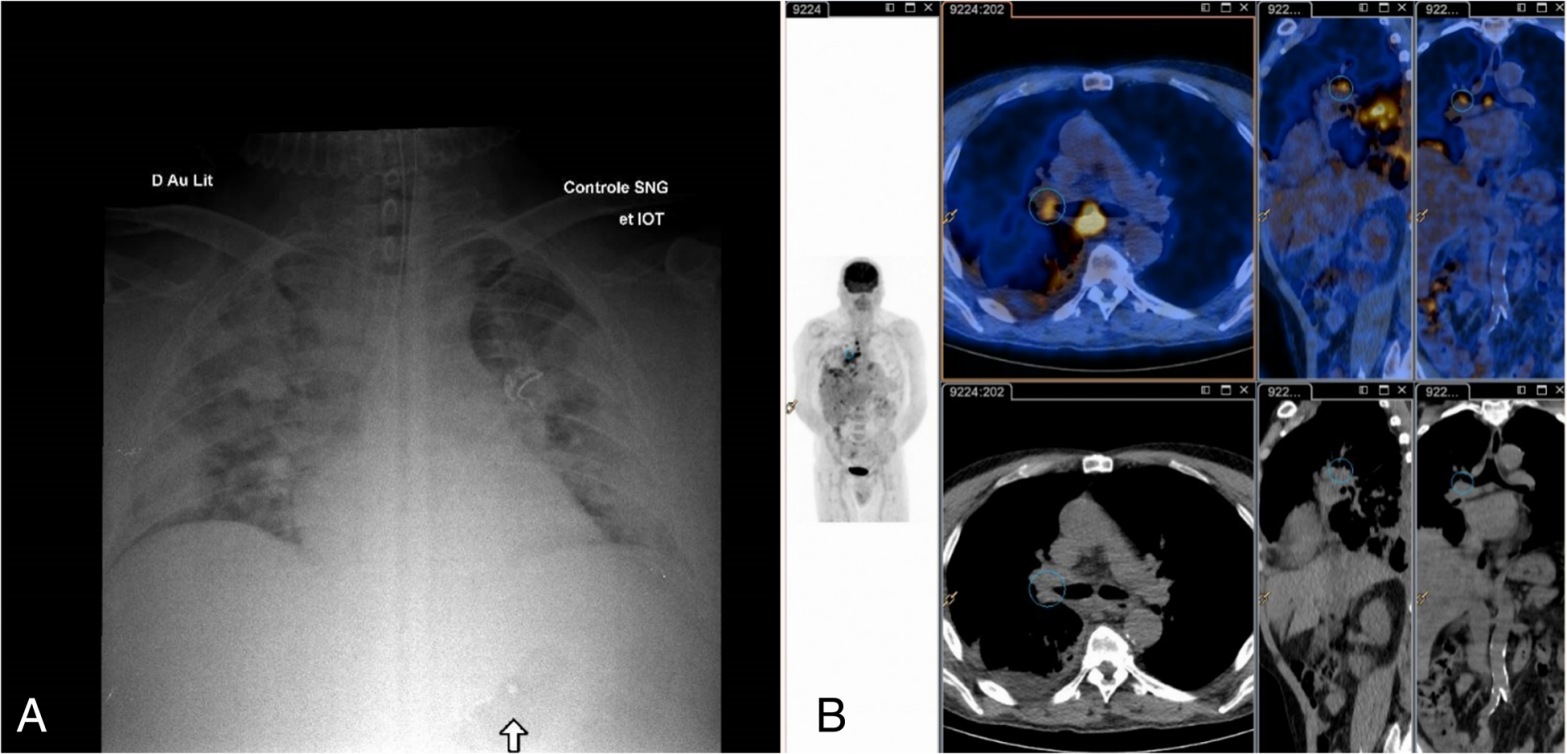
Thoracic imaging showing pulmonary lesions in systemic form of tularemia in a solid organ transplant recipient. **a** Thoracic X-ray showing bilateral alveolar and interstitial opacities during an acute respiratory distress syndrome (Case 2). **b** PET-scan (FDG-F18) showing pulmonary hyperfixations (left inferior lobe) and hypermetabolic mediastinal adenopathies (Case 1)

Blood samples analysis showed leukocytosis with neutrophilia, acute kidney failure and hepatic cytolysis. Chest X-ray showed bilateral alveolar opacities (Fig. 2). Microbiological analyses including *Legionella* and pneumococcal urinary antigens, *Aspergillus* antigenemia, bacterial and fungal culture of bronchio-alveolar aspiration, as well as DNA testing on bronchio-alveolar liquid for *Chlamydophila pneumoniae*, *Legionella pneumophila* and *Mycoplasma pneumonia* were negative. Transthoracic echocardiography showed no sign of endocarditis. Clinical stabilization was obtained by vascular filling and antibiotic therapy with ceftriaxone, spiramycin and two doses of gentamycin. Thoracic CT-Scan showed mediastinal lymphadenopathies and bilateral nodular lesions.

After 5 days, the blood culture finally allowed the recovery of Gram-negative coccobacilli. The standard methods - biochemical and Matrix-assisted laser desorption/ionization time-of-flight MS (MALDI-TOF-MS) on a Vitek-MS instrument (BioMerieux, IVD library) - failed to identify the strain and only 16S-RNA sequencing allowed the identification of this coccobacilli. DNA was extracted from colonies on chocolate polyvitex agar using the Qiagen EZ1 DNA tissue kit according manufacturer’s instructions (Qiagen, Courtaboeuf, France). The 16S rRNA gene was amplified and sequenced with the primer set 27F/16S1RRB, as previously described [9] and bacterial identification was performed using phylogenic analysis on the Bioinformatics Bacterial Identification (BIBI) [10]. The 565 bp sequence obtained for the bacterium to be identified was 100% identical to sequence from *Francisella tularensis* subsp. *holartica*. strain (RNAcentral database accession no. URS00007EECF7). Antimicrobial susceptibility testing showed resistance to β-lactams and sulfamethoxazole/trimethoprim and susceptibility to quinolones and macrolides.

The patient was treated 14 days with ciprofloxacin 500 mg b.i.d with excellent results. Monitoring over 2 years showed full recovery and no further complication or graft rejection event. The patient lived in countryside without any pet. However, he had been cleaning up a vast and dusty barn without wearing any mask and might have inhaled airborne pathogens coming from aerosolization of rodent excrements.

## Discussion and conclusions

We performed an exhaustive review of the literature using PubMed and Google scholar with the following research algorithm: (“*Francisella tularensis*” OR “*holarctica*” OR “tularemia”) AND (“solid organ transplantation” OR “ciclosporin” OR “tacrolimus” OR “transplantation”). No date or language restrictions were applied. Among the 20 results, 7 publications presented cases of tularemia in immunocompromised patients, and 5 cases of tularemia in SOT recipients [11–15]. There were 4 kidney transplant and one liver transplant recipients. Median age at diagnosis of tularemia was 59 years old (range 24–69). One of them presented infection with biovar *tularensis* (patient from the USA), two patients’ biovar was not defined (USA, Turkey) and the others were infected with biovar *holarctica* (USA, France). Although their treatments differed, they all were under immunosuppressive therapy at the time of diagnosis (Table 1).

**Table 1:** Literature review of tularemia in solid organ transplant patients

Reference	Patient characteristics	Type of graft Immunosuppressive therapy	Clinical presentation	Diagnostic method	Antibiotic therapy	Outcome
Limaye & Hooper, 1999 [11]	Male Aged 50. From USA	3 years post-liver transplant for hepatitis C and alcohol related cirrhosis; Prednisolone; Azathioprine.	Fever, arthromyalgia and pneumonia within 72 h.	Blood cultures initially negative came back positive for F*.tularensis* biovar *palearctica* (or holarctica) after 7 days of culture.	Levofloxacin 500 mg/day for 21 days	Complete recovery
Khoury and al, 2005 [12]	Male Aged 69. From USA *(handled hay.)*	4 years post kidney transplant for end-stage renal failure secondary to polycystic kidney disease. Prednisone; Mycophenolate mofetil; Rapamycin	Fever, chills, and neck stiffness associated with fatigue, vomiting, and diarrhea. Patchy infiltrate in the left lung compatible with pneumonia on X-ray	Blood cultures grew *F.tularensis* biovar *tularensis* in 7 days.	Doxycycline for 14 days	Complete recovery
Mittalhenkle A, Norman DJ, 2005 [13]	Age 59 From USA	11 years post kidney transplant secondary to polycystic kidney disease. Prednisone; Mycophenolate mofetil; Cyclosporine	Persistent fever. Chest X-ray and Chest CT-scan showed multiple nodules. BAL and thoracoscopy with nodule biopsy were performed.	Biopsy culture grew a Gram negative bacilli initially identified as *A. actinomycetemcomitans* corrected to *F.tularensis* several days later.	Fluoroquinolone (dosage and duration unknown)	Clinical Improvement
Faucon et al. 2011 [14]	Male Aged 69. From France *(Hunting dog owner.)*	15 years post kidney transplant for IgA nephropathy Prednisolone; Mycophenolate mofetil; Cyclosporine A	Fever, chills, cough and sputum. Chest X-ray showed bilateral interstitial infiltrates.	Blood cultures came back positive for *F.tularensis* biovar *holarctica* after 10 days. PCR confirmed	Levofloxacin 500 mg/day for 14 days	Complete recovery
Ozkok and al, 2012 [15]	Aged 24. From Turkey	12 months after kidney transplant. Prednisolone; Mycophenolate mofetil; Tacrolimus	Cervical lympadenopathy. Pathologic examination of the lymph node showed chronic necrotizing granulomatous inflammation.	Real-time PCR–based test for tularemia performed on the lymph node was positive. Serology later confirmed	Doxycycline 100 mg ×2/day for 4 weeks	Complete recovery

Four of them presented a systemic form with pulmonary involvement and one showed an ulceroglandular form of tularemia. In systemic forms*,* blood cultures were either negative or took several days to grow *F. tularensis* (between 7 and 10 days). Conversely, DNA testing by PCR gave quick and positive results although it was not performed in every case.

In two cases an exposure to aerosolized pathogens was revealed after the diagnosis.

Regarding our 2 cases of systemic tularemia in SOT recipients, respiratory symptoms were at the forefront and the patients showed signs of febrile pneumonia. In each case, the diagnosis was challenging. In the first case, serology and blood cultures were initially negative and only lymph nodes examination including DNA testing on tissue led to the diagnosis. In the second case, blood cultures were positive but standard techniques initially failed to identify *F. tularensis,* thus requiring molecular identification method.

Both the patients received empiric antibiotic therapy for pneumonia that failed to improve their condition but a secondarily adapted antibiotic therapy containing ciprofloxacin proved to be effective. A rigorous questioning revealed an exposure to aerosolized particles in a confined environment in our both cases and in 2 cases from the literature [12, 14]. This highlights the infectious risk for immunocompromised patients in such situations. There is currently no recommendation on the management of a potential exposure for immunocompromised patients. *F. tularensis* is a biosafety level 3 pathogen. Its manipulation in laboratories requires specific conditions including wearing FFP3 masks. However, this level of precaution cannot be reasonably applied to immunocompromised patients in case of environmental exposure. Wearing surgical masks might be a sufficient precaution and educational tips should be given to these patients in order to avoid exposure.

In our two cases and one from literature [12], laboratory technicians were exposed to aerosolized pathogens while performing the different tests required to obtain a diagnosis. They all received a prophylactic treatment with Doxycycline or Ciprofloxacin and no complication was reported. Nonetheless, these situations could have been avoided with standard precautions by informing the lab staff of this possible diagnosis after clinical examination and anamnesis. This highlights the risks of delaying diagnosis both for patients and laboratory staff.

Tularemia is a rare disease and only 5 cases in SOT recipients were reported earlier. This study highlights a frequent pulmonary presentation in such situation and suggests that it should be considered in case of pneumonia in a SOT recipient with a history of potential exposure to aerosolized pathogens.

The fastidiousness of culture recovery of *F. tularensis* and its potential dissemination underline the need for a faster identification. Results of serology often take time to be obtained and control is often necessary. Thus, the quick and positive results obtained in the different cases with molecular techniques (universal 16S-RNA PCR or specific *F. tularensis* PCR) on pathological tissues suggest that these techniques should be considered when tularemia is suspected in SOT recipient. Even though the molecular techniques on Bronchio-Alveolar Lavage fluids might appear as a diagnostic tool for systemic form of Tularemia, their interest have not been described in the literature. The lack of study on this diagnostic approach is mainly explained by the rareness of this disease. Further data on the effectiveness of this procedure might provide valuable information for the physicians taking care of immunocompromised patients.

In conclusion, Tularemia should be considered in SOT recipients presenting unexplained fever and clinical or radiological pulmonary symptoms. Thorough interrogatory looking for potential inhalation of pathogens is necessary and should be conducted. Simple precaution tips should be given to immunocompromised patients such as wearing masks in critical situations. In certain cases, molecular techniques on pathological tissues might improve diagnosis with faster results.

## Abbreviations

ANA: Antinuclear antibodies
ANCA: Anti-neutrophil cytoplasmic antibodies
BAL: Bronchio-Alveolar Lavage
CT-scan: Computed Tomography scan
DNA: Desoxyribonucleic acid
FFP3: Filtering Facepiece Particles type 3
ICU: Intensive care unit
MRI: Magnetic resonance imaging
PCR: Polymerase chain reaction
PET-scan: Positon Emission tomography scan
RNA: Ribonucleic acid
SOT: Solid organ transplant
